# Investigating the role of eight SNPs in *CHRNA3* for COPD susceptibility in the Chinese elderly population

**DOI:** 10.1080/07853890.2025.2474726

**Published:** 2025-03-12

**Authors:** Yamei Zheng, Jie Zhao, Meihua Liu, Yunchan Liu, Yipeng Ding, Tian Xie

**Affiliations:** Department of Respiratory and Critical Care Medicine, Hainan General Hospital, Hainan Affiliated Hospital of Hainan Medical University, Haikou, Hainan, China

**Keywords:** COPD, *CHRNA3*, SNPs, elderly, genetic susceptibility

## Abstract

**Background:**

Chronic obstructive pulmonary disease (COPD) is a leading cause of morbidity and mortality among the elderly in China. Genetic predisposition is a recognized risk factor for COPD, with *CHRNA3* emerging as a promising candidate gene due to its involvement in smoking behavior and lung function. This study aimed to investigate the association between eight *CHRNA3* SNPs and COPD susceptibility in the Chinese elderly population.

**Methods:**

A total of 270 COPD patients and 271 healthy controls were included in the study. SNP genotyping was carried out using the Agena MassARRAY platform. Logistic regression analysis was employed to calculate odds ratios (ORs) and 95% confidence intervals (CIs) to assess the association between the SNPs and COPD risk. Forest plots were generated using Sangerbox software to visually represent the association results. Additionally, haplotype blocks were constructed using Haploview 4.2 software to explore the potential impact of haplotypes on COPD risk.

**Results:**

Our findings indicated that rs615470, rs660652, and rs472054 are associated with a reduced risk of COPD, while rs8040868 is associated with an increased risk. Linkage disequilibrium (LD) analysis identified a haplotype block encompassing rs76071148, rs615470, rs660652, rs472054 and rs578776. Notably, the haplotype TTAAG was associated with a reduced risk of COPD.

**Conclusion:**

This study provides valuable insights into the genetic susceptibility of COPD among the elderly, particularly regarding the role of SNPs in *CHRNA3*. These findings contribute to a deeper understanding of the pathogenesis of COPD and may facilitate the discovery of novel therapeutic targets for COPD.

## Introduction

Chronic obstructive pulmonary disease (COPD) is a chronic inflammatory condition of the lungs, characterized by persistent airflow limitation that is often caused by exposure to harmful particles or gases [1]. This debilitating illness disproportionately affects the elderly population due to their longer history of smoking and cumulative exposure to other environmental factors, making it a significant public health concern worldwide [2,3]. Age-related changes in lung function further increase the susceptibility of the elderly to COPD [4]. The etiology of COPD is complex, encompassing both genetic and environmental factors [5]. While smoking [6] remains the primary modifiable risk factor, the role of genetics [7] in determining individual susceptibility to COPD is becoming increasingly apparent. Genetic variations, specifically single nucleotide polymorphisms (SNPs), can affect gene expression or function. These variations can modulate an individual’s response to environmental triggers, thereby influencing COPD susceptibility [8,9].

*CHRNA3* is a gene encoding nicotinic acetylcholine receptors (nAChRs), transmembrane proteins expressed in various tissues, including the brain and lungs. These receptors function as ligand-gated ion channels and play a pivotal role in neurotransmission, inflammation, and apoptosis [10–13]. I in the context of COPD, nAChRs are expressed in the airways and become activated by cigarette smoke, triggering airway inflammation and remodeling [14]. SNPs in *CHRNA3* have the potential to modify the function of these receptors, potentially contributing to COPD susceptibility. Previous studies examining the association between SNPs in *CHRNA3* and COPD risk have yielded conflicting results. Some studies have reported significant associations, while others have failed to detect any significant association [15–17]. These inconsistencies may be attributed to differences in study populations, sample sizes, genotyping methods, and other confounding factors. Given the significance of COPD among the elderly population and the inconsistent findings, it is imperative to further investigate the role of SNPs in *CHRNA3* in determining COPD susceptibility within this specific group.

**Figure 1.**
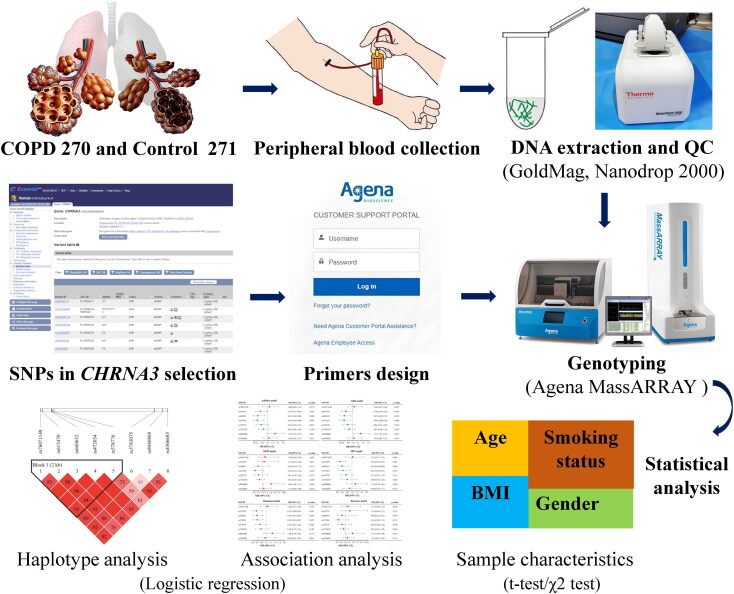
Detailed flowchart of the research process. *Abbreviations*: COPD: chronic obstructive pulmonary disease; QC: quality control.

Therefore, the current study aims to delve into the association between eight SNPs in *CHRNA3* and COPD susceptibility among the elderly Chinese population. Please refer to Figure 1 for a visual representation of the research flowchart. Through examining the influence of these SNPs in this particular group, we seek to gain a more profound comprehension of the genetic factors that underlie COPD risk. The insights gained from this study have the potential to guide future research endeavors and enhance the prevention and treatment of COPD among the elderly.

## Methods

### Study population

A total of 270 patients diagnosed with COPD and 271 healthy control subjects were recruited from Hainan General Hospital for this study. The inclusion and exclusion criteria for participants were as follows: Inclusion criteria: All patients were diagnosed with COPD based on their clinical history, physical examination, and spirometry findings (e.g. a post-bronchodilator forced expiratory volume in the first second (FEV1)/forced vital capacity (FVC) ratio <0.70). These patients had to have had a stable COPD condition for at least three months prior to enrollment. Control subjects were those with no history of COPD or other respiratory diseases, such as pulmonary fibrosis, bronchiectasis, or asthma. Exclusion criteria: Subjects were excluded if they were using long-term inhaled corticosteroids or other medications that could potentially affect lung function. The subjects’ ages ranged from 40 to 95 years. Additionally, personal information, including age, sex, weight, height, ethnicity, and smoking history, was collected. Subjects were categorized as smokers or non-smokers based on their smoking history. Body Mass Index (BMI) was calculated as weight (in kilograms) divided by height (in meters) squared.

The study was approved by the Ethics Committee of Hainan General Hospital (No. [2022]-312), and all procedures were conducted in accordance with the Declaration of Helsinki. All subjects were informed about the study aims and procedures, and written informed consent was obtained from each participant.

### DNA extraction

Genomic DNA was isolated from whole blood samples collected from COPD patients and healthy controls using the GoldMag Mini Whole Blood Genomic DNA Purification Kit (GoldMag. Co., Ltd., Xi’an, China). Subsequently, the quality and quantity of the extracted DNA were evaluated using a Nanodrop 2000 spectrophotometer (Thermo Fisher Scientific, Waltham, MA, USA). The minimal concentration prerequisite for acceptable DNA samples is set at 20 ng/μl. Additionally, the DNA purity, indicated by the optical density ratio (OD260/280), must fall within the acceptable range of 1.7–2.0 to ensure purity.

### Selection and genotyping of SNPs

In selecting SNPs for analysis, we prioritized those located in the *CHRNA3* gene with a minor allele frequency (MAF) exceeding 0.05 in the global population. This criterion was obtained from the 1000 Genomes Project database (GRCh38) and ensures genetic diversity, encompassing a wide range of allelic variations. This, in turn, enhances the statistical power and reliability of our genotyping analysis. Additionally, we referred to previously published articles and randomly selected eight SNPs in *CHRNA3*, including rs76071148, rs615470, rs660652, rs472054, rs578776, rs3743075, rs8040868, rs4366683 [15,18–22]. The primer sequences outlined in Table 1 were meticulously designed using the online software, Agena Bioscience Assay Design Suite Version 2.0. Bioengineering (Shanghai Co., Ltd, China) was entrusted with the synthesis of these primers. To ensure precise genotyping of the eight SNPs in *CHRNA3*, the Agena MassARRAY platform from Agena Bioscience (San Diego, CA, USA) was employed. Furthermore, the Agena Bioscience TYPER software (version 4.0) was utilized for data management, analysis, and accurate interpretation of the genotyping results. These measures combined ensure reliability and precision throughout the genotyping process.

**Table 1.** Primer sequences for PCR and unique extension reaction.

SNP-ID	Forward of PCR (5′–3′)	Reverse of PCR (5′–3′)	Unique extension (5′–3′)
rs76071148	ACGTTGGATGAATTGTTGGATCTCTTGGGC	ACGTTGGATGGGGAGGCTTCACTTATTTGC	cccGGCAAATATATTAATACCAGTTCA
rs615470	ACGTTGGATGCTGCATTTGGTAAAGGTATG	ACGTTGGATGATTATATCAAGTAGTCAGC	gAAAGGTATGTAAACTTTCCTG
rs660652	ACGTTGGATGATGCCAATGACATACCTTGC	ACGTTGGATGAACCAGGGGCAATTTTGCTC	ctcATGTCTCCATTGTTAAATGT
rs472054	ACGTTGGATGTTTGGACCACAGCATGAACC	ACGTTGGATGTACCCCCATTCTGTACCAAC	TCTCAGCCTTAGCACTA
rs578776	ACGTTGGATGACTGGGTCTAAAGGGCTATG	ACGTTGGATGCAATGAATAACTAGGCATGA	gCTCTTGCATACTTCTAAATTATAC
rs3743075	ACGTTGGATGCCAGGCTGATTCTTTTACCG	ACGTTGGATGAACTCTGCCCCACCATAGTC	acCTGGAATGACTACAAGCTGAA
rs8040868	ACGTTGGATGTCTATTTGAGCGGCTGTTTG	ACGTTGGATGTGGACACCTCGAAATGGATG	ggggaAATGAGATCATCCGGCCTGT
rs4366683	ACGTTGGATGGAATCCAACTTCCTCAGGTC	ACGTTGGATGCCTGGGACCCTTCTTTCATC	aacctAAATAGGATTTTTCCTGCTCCC

SNP: single nucleotide polymorphism; PCR: polymerase chain reaction.

### Statistical analyses

In order to assess the relationship between genetic variants and COPD susceptibility, a comprehensive statistical analysis was conducted. Initially, the distributions of continuous and categorical variables were compared between case and control groups using the independent sample *T*-test and Pearson’s Chi-Square test, respectively. To ensure the validity of the genetic data, Hardy–Weinberg equilibrium (HWE) was assessed for genotype frequencies of the eight SNPs in *CHRNA3* in the control group using the chi-square test. Subsequently, the association between eight SNPs in *CHRNA3* and COPD susceptibility was evaluated based on the calculation of odds ratios (ORs) and 95% confidence intervals (CIs) using logistic regression analysis. Multiple genetic models were considered, including allele, codominant, dominant, recessive, and additive models. To ensure accurate results, the analysis was adjusted for factors such as age, gender, body mass index (BMI), and smoking status. To visualize the results of these analyses, forest plots were generated using the Sangerbox software (http://sangerbox.com/home.html).

Moreover, Haploview 4.2 software was used to construct haplotype blocks based on linkage disequilibrium (LD) patterns. LD, measured by the D’ statistic, represents the non-random association between alleles at different loci. A D’ value close to 1 indicates strong LD, indicating that the alleles at the two loci tend to be inherited together. Conversely, a D’ value close to 0 suggests no LD, indicating that the alleles are inherited independently. Haplotype analysis was employed to identify haplotypes that are significantly associated with COPD risk using the SNPStats online software (https://snpstats.net/analyzer.php). Microsoft Excel (Microsoft Corp., Redmond, WA, USA) was utilized for basic data manipulation and preliminary analysis. Advanced statistical analysis, including logistic regression and chi-square tests, was performed using the Statistical Package for the Social Sciences (SPSS) version 20 (SPSS, Chicago, IL). Additionally, PLINK software (version 1.07) was employed for genetic data analysis, including Hardy–Weinberg equilibrium testing and SNP association analysis. A statistically significant difference is indicated when the *p*-value is less than 0.05.

## Results

The results of this comparative analysis of sample characteristics between the case group and the control group are summarized in Table 2. Firstly, a significant difference was observed in mean age between the two groups. Specifically, the mean age of the case group was 72.26 ± 10.40 years, which was notably higher than the mean age of 69.21 ± 6.61 years in the control group (*p* < 0.001). This finding aligns with the known epidemiology of COPD, which typically affects older individuals. However, the difference in gender distribution between the case group (76.3% male) and the control group (77.5% male) was not statistically significant (*p* = 0.742). Additionally, there was no significant difference in mean BMI between the case group (21.35 ± 3.66 kg/m^2^) and the control group (21.64 ± 3.70 kg/m^2^), with a *p*-value of 0.350. Finally, we compared the smoking status of participants in both groups, this difference was not statistically significant (*p* = 0.518). Collectively, the results of this comparative analysis demonstrate that the case and control groups were well-matched in terms of gender, smoking status, and BMI, minimizing the potential confounding effects of these variables on the association between SNPs in *CHRNA3* and COPD susceptibility.

**Table 2.** Comparative analysis of sample characteristics between case group and control group.

Characteristics		Case (*n* = 270)	Control (*n* = 271)	*p*-Value
Age (Mean ± SD, Years)		72.26 ± 10.40	69.21 ± 6.61	<0.001
Gender	Male	206 (76.3%)	210 (77.5%)	0.742
	Female	64 (23.7%)	61 (22.5%)	
BMI (Mean ± SD, kg/m^2^)		21.35 ± 3.66	21.64 ± 3.70	0.350
Smoking status	Smoker	142 (52.6%)	135 (49.8%)	0.518
	Non-smoker	128 (47.4%)	136 (50.2%)	

SD: standard deviation; BMI: body mass index.

For the comparison of continuous variables, Student’s *t*-test was utilized, whereas categorical variables were compared using the *χ*^2^ test.

*p*-Value <0.05 is considered statistically significant.

Table 3 shows the basic information of eight SNPs in *CHRNA3*, including their chromosome position, minor/major allele, functional annotation, MAF in both the case and control groups, and the HWE *p*-value. The genotype frequency is shown in Supplementary Figure 1. All SNPs with HWE-*p* values greater than 0.05 suggest that these markers are genetically equilibrated within the sampled population. This equilibrium state implies that the allele and genotype frequencies of these SNPs are stable and unlikely to have been significantly altered by processes such as non-random mating, mutations, population migrations, or natural selection. However, it does not guarantee the absence of any association between these SNPs and COPD risk. Therefore, further genetic association analyses are required to assess their potential association between these SNPs in *CHRNA3* and susceptibility to COPD.

**Table 3.** Information on eight SNP in the *CHRNA3* gene.

SNP-ID	Chromosome position	Alleles A/B	Functional annotation	MAF-case	MAF-control	HWE-*p*
rs76071148	Chr15:78593232	T/A	Intronic variant	0.269	0.256	0.874
rs615470	Chr15:78593646	T/C	Intronic variant	0.170	0.218	0.595
rs660652	Chr15:78595490	A/G	Intronic variant	0.154	0.212	0.149
rs472054	Chr15:78595652	A/G	Intronic variant	0.149	0.210	0.199
rs578776	Chr15:78596058	G/A	3′UTR variant	0.207	0.244	0.247
rs3743075	Chr15:78617110	T/C	Missense variant	0.435	0.470	0.542
rs8040868	Chr15:78618839	T/C	5′UTR variant	0.398	0.336	0.785
rs4366683	Chr15:78619861	C/T	5′UTR variant	0.502	0.489	0.182

SNP: single nucleotide polymorphism; Chr: chromosome; A/B: minor/major allele; MAF: minor allele frequency; HWE: Hardy–Weinberg equilibrium; UTR: untranslated region.

The HWE-*p* values were determined through the *χ*^2^ test, with statistical significance set at *p* < 0.05.

**Figure 2.**
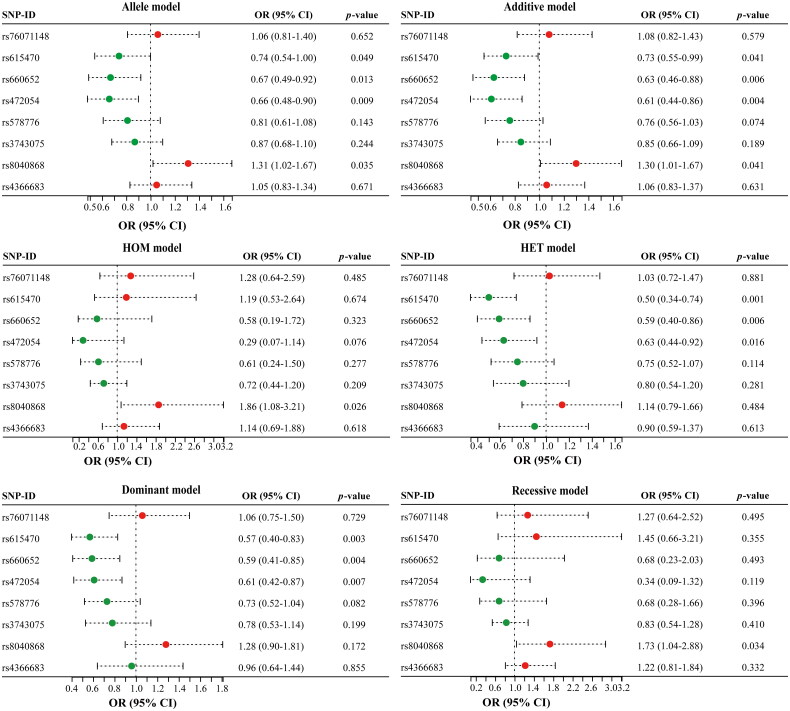
Forest plot of *CHRNA3* SNPs associations with COPD under various genetic models. The allele model displays the OR and 95% CI for the effect of each allele. The additive model posits that the effect of the genetic variant is proportional to the number of copies of the effect allele an individual possesses. The HOM model compares the OR of individuals homozygous for the variant allele to those homozygous for the reference allele. The HET model compares the OR of heterozygous individuals to those homozygous for the reference allele. The dominant model’s OR compares individuals with at least one copy of the effect allele to those without it. The recessive model’s OR compares individuals homozygous for the effect allele to all other genotypes combined. *Abbreviations*: SNP: single nucleotide polymorphism; OR: odds ratio; CI: confidence interval; HOM: homozygous; HET: heterozygote. *p* < 0.05 was considered to be significant.

The forest plots presented in Figure 2 illustrated the association between SNPs in the *CHRNA3* gene and susceptibility to COPD in the elderly. For rs615470 in *CHRNA3*, the allele model showed a significant association between the T allele and reduced risk of COPD (OR = 0.74, 95% CI: 0.54–1.00, *p* = 0.049). The codominant model revealed a stronger association, with individuals carrying the C/T genotype having a significantly lower risk of COPD (OR = 0.50, 95% CI: 0.34-0.74, *p* = 0.001). The dominant model also demonstrated a significant association, with a combined C/T-T/T genotype associated with reduced risk of COPD (OR = 0.57, 95% CI: 0.40-0.83, *p* = 0.003). Under the additive model, rs615470 also exhibited a significant association with a reduced risk of COPD (OR = 0.73, 95% CI: 0.55-0.99, *p* = 0.049). However, the recessive model did not show significant associations.

Similarly, the allele model indicated a significant association between the A allele of the two SNPs (rs660652 and rs472054) in *CHRNA3* and reduced risk of COPD (OR = 0.67, 95% CI: 0.49–0.92, *p* = 0.013; OR = 0.66, 95% CI: 0.48–0.90, *p* = 0.009, respectively). The codominant and dominant models also showed significant associations, with individuals carrying the G/A or A/A genotypes of rs660652 (OR = 0.59, 95% CI: 0.40–0.86, *p* = 0.006 and OR = 0.59, 95% CI: 0.41–0.85, *p* = 0.004, respectively) and rs472054 (OR = 0.63, 95% CI: 0.44–0.92, *p* = 0.016 and OR = 0.61, 95% CI: 0.42–0.87, *p* = 0.007, respectively) having a lower risk of COPD compared to those with the G/G genotype. The recessive model did not reach significance, while the additive model also demonstrated a strong association between rs660652 (OR = 0.63, 95% CI: 0.46–0.88, *p* = 0.006) and rs472054 (OR = 0.61, 95% CI: 0.44–0.86, *p* = 0.004) and susceptibility to COPD.

Additionally, we discovered a strong association between rs8040868 in *CHRNA3* and an elevated risk of developing COPD. Specifically, our findings indicated that the allele C carried a 1.31-fold increased risk of COPD compared to the reference allele T (OR = 1.31, 95% CI: 1.02–1.67, *p* = 0.035). This association was further strengthened under the codominant model, where the C/C genotype was significantly associated with increased risk of COPD (OR = 1.86, 95% CI: 1.08–3.21, *p* = 0.026). The recessive model also demonstrated a significant association between the C/C genotype and an increased risk of COPD (OR = 1.73, 95% CI: 1.04–2.88, *p* = 0.034). Additionally, the additive model confirmed this association, indicating a 1.30-fold increased risk of COPD for each additional C allele (OR = 1.30, 95% CI: 1.01–1.67, *p* = 0.041). However, no significant associations were observed between rs76071148, rs578776, and rs3743075 in *CHRNA3* and the risk of COPD.

**Figure 3.**
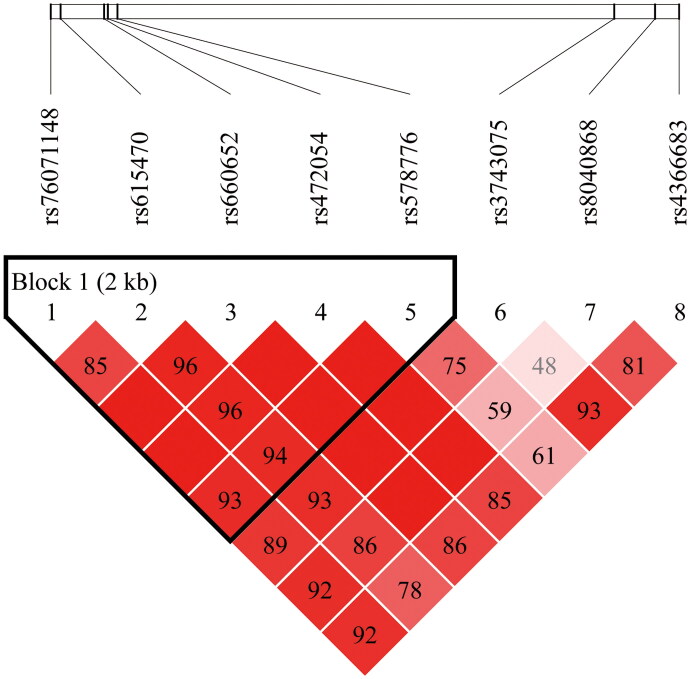
The linkage disequilibrium patterns among SNPs in *CHRNA3* and the haplotype blocks. This heatmap illustrates the LD among SNPs in the *CHRNA3* gene, indicating how often certain alleles are inherited together. The matrix uses a color gradient to represent the strength of LD between SNPs, with deeper colors signifying higher LD and lighter colors indicating weaker LD. Defined regions within the genome are characterized by a high degree of LD among neighboring SNPs, suggesting they are less likely to be separated by recombination events. Numerical values/100 superimposed on the heatmap blocks are D’ values. Values close to 1 indicate a strong association between alleles, while values close to 0 suggest little to no association. *Abbreviations*: LD: Linkage disequilibrium.

To further investigate the association between these SNPs and the risk of COPD, we conducted LD and haplotype analysis. LD analysis identified a haplotype block including rs76071148, rs615470, rs660652, rs472054 and rs578776, as demonstrated by D’ statistics of Figure 3. The haplotype TTAAG was associated with a reduced risk of COPD (OR = 0.60, 95% CI: 0.42–0.87, *p* = 0.007) compared to the reference haplotype TCGGA, as shown in Table 4.

**Table 4.** Association between haplotypes in *CHRNA3* and susceptibility to COPD.

SNP-ID	Haplotype	Control-Fre	Case-Fre	OR (95% CI)	*p*-Value
rs76071148/rs615470/rs660652/rs472054/rs578776	TCGGA	0.498	0.504	1.000	---
	ACGGA	0.248	0.258	1.02 (0.75–1.39)	0.900
	TTAAG	0.205	0.138	0.60 (0.42–0.87)	0.007
	TCGGG	0.028	0.051	1.56 (0.77–3.15)	0.220
	TTGGA	0.004	0.022	3.75 (0.79–17.87)	0.097
	rare	0.018	0.027	1.14 (0.41–3.17)	0.800

SNP: single nucleotide polymorphism; Fre: frequency; OR: odds ratio; CI: confidence interval.

*p* < 0.05 was considered to be significant.

## Discussion

COPD, a chronic inflammatory disease of the lungs, represents a significant health burden in the elderly population. Genetic factors, including SNPs, play a crucial role in determining individual susceptibility to this condition. In the present study, we investigated the association between eight SNPs in *CHRNA3* and COPD susceptibility in the elderly. Our findings revealed that rs615470, rs660652, and rs472054 were associated with a reduced risk of COPD in the Chinese elderly population, indicating that these SNPs may confer protective effects against the disease. On the other hand, rs8040868 was associated with an increased risk of COPD, suggesting that it may contribute to the pathogenesis of the disease. In addition to single SNP analysis, we also conducted haplotype analysis to explore the potential impact of haplotypes on COPD risk. LD analysis identified a haplotype block including rs76071148, rs615470, rs660652, rs472054, and rs578776. Notably, the haplotype TTAAG was associated with a reduced risk of COPD. This finding suggests that haplotypes in *CHRNA3* may contribute to the genetic susceptibility of COPD and that combinations of SNPs may have a stronger effect on disease risk than individual SNPs.

Multiple studies have investigated the association between *CHRNA3* and COPD, focusing on genetic polymorphisms that may influence disease susceptibility. for example, *CHRNA3* SNP rs1051730 has been shown to be significantly associated with an increased risk of COPD [23,24]. Additionally, the allele of rs8040868 in *CHRNA3* has been associated with an increased risk of COPD [22]. Previous study has demonstrated that the CC genotype of rs8040868 indicates a higher risk for lung cancer among non-smokers [25]. On the other hand, the (CT + CC) genotype of rs8040868 exerts a protective effect against severe emphysema [18]. Our study also demonstrates a significant association between the allele C and CC genotype of rs8040868 and an increased risk of COPD in the elderly. However, it’s important to note that genetic associations with COPD are complex and may vary across different ethnic groups. For instance, our study has identified the association between rs660652 and COPD risk in the Chinese elderly population, previous studies in the Korean population did not reveal the same association [15]. This highlights the need for further research to understand the ethnic differences in genetic polymorphisms and their effects on COPD susceptibility. Furthermore, the association of rs660652 with smoking cessation indicates a potential involvement of these variants in nicotine dependence and smoking behaviors [26]. This suggests that rs660652 may play a role in the development of COPD by modulating smoking-related processes, including nicotine dependence and smoking cessation.

Despite the current study’s failure to found an association between rs76071148, rs3743075, and rs578776 with COPD susceptibility in the Chinese elderly population, previous studies have identified various association between these SNPs and pulmonary function as well as smoking cessation. Specifically, carriers of the rs76071148 AT genotype were found to have a reduced likelihood of developing severe emphysema [18]. Additionally, rs3743075 in *CHRNA3* was significantly associated with the Fagerström Test for Nicotine Dependence score[21]. Furthermore, the SNP rs578776 in *CHRNA3* was associated with an increased probability of smoking cessation among adult women [27] as well as smoking status in a Bangladeshi population [28]. Notably, smokers with the AA genotype of rs578776 exhibited a strong protective effect against the development of nicotine dependence in the Bangladeshi population [20]. Moreover, rs578776 was found to be associated with lung cancer risk [24]. Given that smoking cessation has been shown to slow the progression, improve symptoms, and decrease mortality rates of COPD [29,30], these genetic findings offer further insights into the multifaceted etiology of the disease. Meanwhile, we found that rs615470 and rs472054 were associated with a decreased risk of COPD among the elderly, while no significant association was observed for rs4366683 in the elderly population. Reports have shown that the interaction between rs615470 and recent smoking may affect cognitive flexibility [19]. Our findings regarding the variants (rs615470, rs472054, and rs4366683) are novel and contribute to the evolving understanding of COPD genetics.

While the findings provide valuable insights, several limitations need to be acknowledged. Firstly, the study population was limited to a specific ethnic group, which may limit the generalizability of the findings to other populations. Future studies should aim to include a more diverse sample of participants to assess the association between these SNPs and COPD susceptibility across different ethnic and genetic backgrounds. Secondly, the current investigation did not delve into the functional repercussions of the SNPs concerning gene expression or protein functionality. It is imperative for future research to conduct functional analyses to clarify the biological processes that link these SNPs to COPD susceptibility. To this end, we plan to employ methodologies such as luciferase reporter assays, chromatin immunoprecipitation (ChIP), and RNA interference (RNAi) for both *in vitro* and *in vivo* functional assessments in upcoming studies. These will help us to scrutinize how the identified SNPs might modulate gene expression and protein activity. Moreover, we intend to amalgamate our results with existing multi-omics datasets, including transcriptomics, proteomics, and metabolomics. This synthesis will facilitate the identification of the SNPs’ potential downstream impacts on biological pathways implicated in COPD, thereby enabling us to discern the precise mechanisms through which these SNPs may enhance disease vulnerability. Thirdly, the study focused primarily on SNPs in *CHRNA3* and did not explore other potential genetic loci or genes that may be involved in COPD susceptibility. Lastly, while we focused on SNPs in *CHRNA3*, COPD susceptibility is likely influenced by multiple genes and environmental factors. Future studies incorporating a more comprehensive genetic approach and larger sample sizes are needed to confirm our findings and identify additional genetic variants associated with COPD risk.

## Conclusion

The overall findings indicate that SNPs rs615470, rs660652, and rs472054 in the *CHRNA3* gene may be associated with a decreased risk of COPD in the elderly, whereas rs8040868 is associated with an elevated risk. Notably, SNPs rs76071148, rs578776, and rs3743075 did not exhibit significant associations with COPD. These observations could significantly contribute to our understanding of the genetic predisposition towards COPD in the elderly and potentially aid in the discovery of novel therapeutic targets.

## Supplementary Material

Supplemental Material

## Data Availability

All data included in this study are available upon request by contact with the corresponding author.
