# Supplementary material for: Investigating the role of eight SNPs in CHRNA3 for COPD susceptibility in the Chinese elderly population
Source: Ann Med. 2025 Mar 12;57(1):2474726. doi: 10.1080/07853890.2025.2474726 (PMC11905312; doi:10.1080/07853890.2025.2474726)

Supplement Figure 1 The genotype frequencies of eight SNPs in CHRNA3 in the case and control groups

The chart uses color-coded bars to represent different genotypes for each SNP. AA: Homozygous mutant genotype, depicted by a blue bar. AB: Heterozygous mutant genotype, depicted by an orange bar. BB: Homozygous wild-type genotype, depicted by a gray bar. The y-axis represents the percentage of individuals within each group who possess a particular genotype, while the x-axis lists the different SNPs. The numbers displayed on the bars indicate the exact percentage of individuals in each group with the corresponding genotype


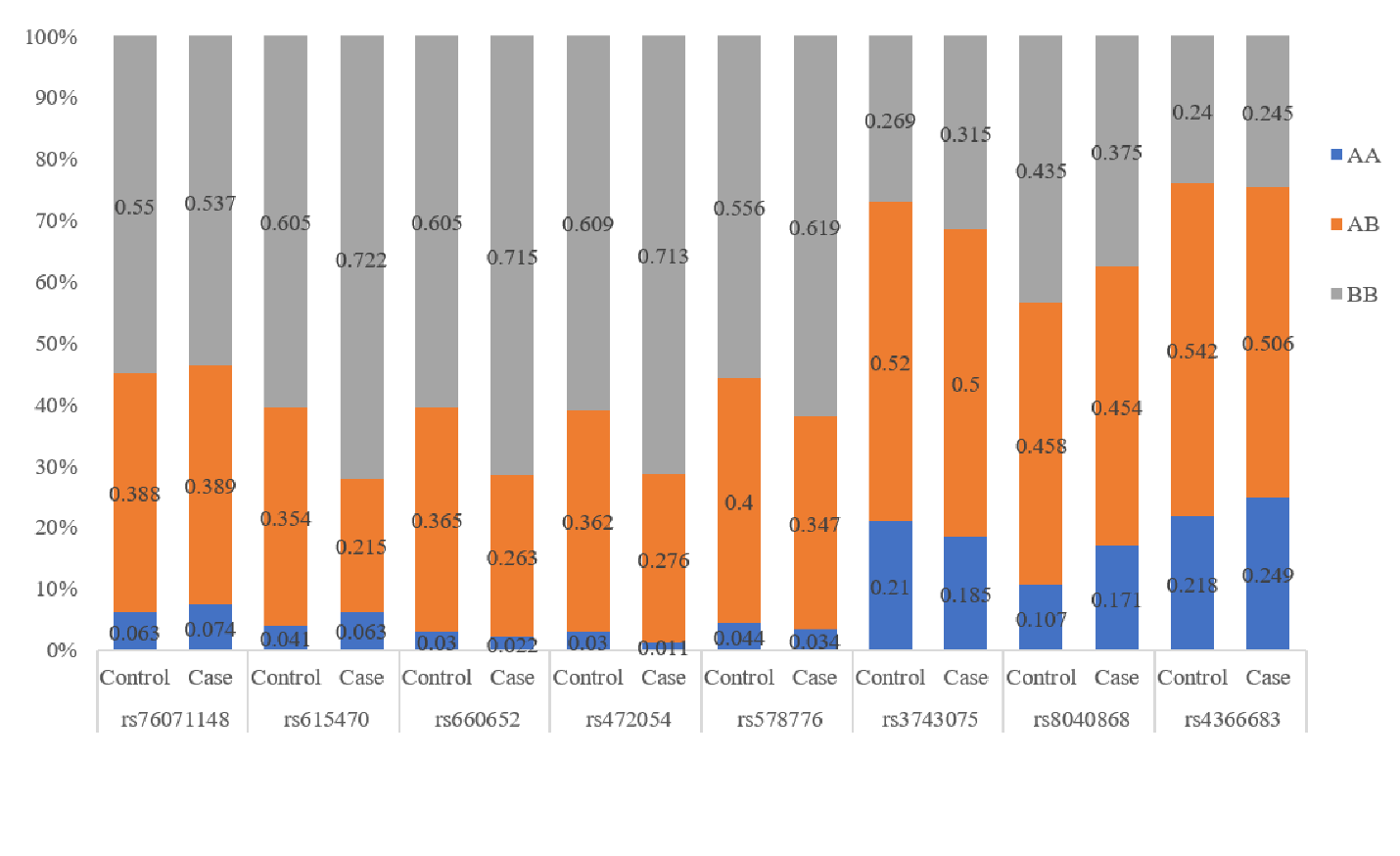

Supplement: Supplemental Material [file IANN_A_2474726_SM4644.docx]
